# Emerging Therapies for Hepatocellular Carcinoma (HCC)

**DOI:** 10.3390/cancers14112798

**Published:** 2022-06-04

**Authors:** Eesha Chakraborty, Devanand Sarkar

**Affiliations:** 1 C. Kenneth and Dianne Wright Center for Clinical and Translational Research, Virginia Commonwealth University, Richmond, VA 23298, USA; chakrabortye@vcu.edu; 2 Department of Human and Molecular Genetics, Massey Cancer Center, VCU Institute of Molecular Medicine (VIMM), Virginia Commonwealth University, Richmond, VA 23298, USA

**Keywords:** HCC, immunotherapy, gene therapy, CRISPR/Cas9, (CAR)-T cells, oncolytic virus, PD-1, PD-L1, nanoparticle, clinical trials

## Abstract

**Simple Summary:**

Primary liver cancer, also known as Hepatocellular carcinoma (HCC), is considered to be a major global health challenge. Due to delays in diagnosis at early asymptomatic stages, HCC reaches a severe aggressive stage, thereby having a significant negative impact on patient survival. In addition, HCC shows marked resistance to conventional cancer treatments such as chemo- and radiotherapy. A variety of new and advanced therapies are continuously being evaluated to acquire a breakthrough in HCC treatment to enhance overall and recurrence-free survival. Appropriate identification and selection of target genes and utilization of safe and effective therapeutic approaches, such as gene therapy or immunotherapy, are key strategies for the effective treatment for HCC. This review paper intends to provide a perspective on emerging approaches as avenues towards more effective and safer therapies for HCC.

**Abstract:**

Hepatocellular carcinoma (HCC) arises from hepatocytes and accounts for 90% of primary liver cancer. According to Global Cancer Incidence, Mortality and Prevalence (GLOBOCAN) 2020, globally HCC is the sixth most common cancer and the third most common cause of cancer-related deaths. Reasons for HCC prognosis remaining dismal are that HCC is asymptomatic in its early stages, leading to late diagnosis, and it is markedly resistant to conventional chemo- and radiotherapy. Liver transplantation is the treatment of choice in early stages, while surgical resection, radiofrequency ablation (RFA) and trans arterial chemoembolization (TACE) are Food and Drug Administration (FDA)-approved treatments for advanced HCC. Additional first line therapy for advanced HCC includes broad-spectrum tyrosine kinase inhibitors (TKIs), such as sorafenib and lenvatinib, as well as a combination of immunotherapy and anti-angiogenesis therapy, namely atezolizumab and bevacizumab. However, these strategies provide nominal extension in the survival curve, cause broad spectrum toxic side effects, and patients eventually develop therapy resistance. Some common mutations in HCC, such as in telomerase reverse transcriptase (*TERT*), catenin beta 1 (*CTNNB1*) and tumor protein p53 (*TP53*) genes, are still considered to be undruggable. In this context, identification of appropriate gene targets and specific gene delivery approaches create the potential of gene- and immune-based therapies for the safe and effective treatment of HCC. This review elaborates on the current status of HCC treatment by focusing on potential gene targets and advanced techniques, such as oncolytic viral vectors, nanoparticles, chimeric antigen receptor (CAR)-T cells, immunotherapy, and clustered regularly interspaced short palindromic repeats/CRISPR-associated protein 9 (CRISPR/Cas9), and describes future prospects in HCC treatment.

## 1. Introduction

**Figure 1:**
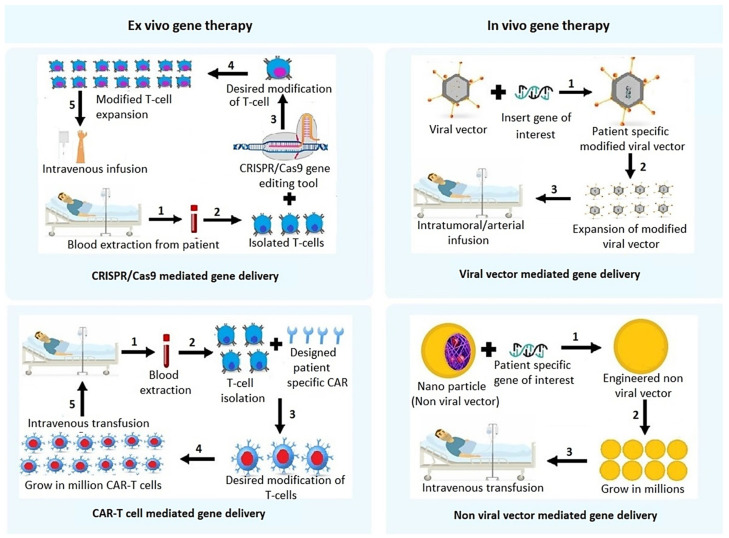
An overview of the gene- and immune-based therapeutic techniques for HCC.

Hepatocellular carcinoma (HCC), arising from hepatocytes, accounts for around 90% of primary liver cancer. The occurrence of HCC is commonly observed in males to a greater extent as compared to females. Geographical variation also has a significant contribution to HCC onset with a majority of cases occurring in Asia. Major known risk factors associated with HCC are viral (chronic hepatitis B and C), metabolic (diabetes and non-alcoholic fatty liver disease or NAFLD), toxic (alcohol and aflatoxins) and immune system-related disorders. HCV patients with baseline liver stiffness show correlation in developing HCC [1]. However, direct acting antiviral (DAA)-mediated induction of sustained virologic response (SVR) reduces the risk of HCC in the mid-long term [2]. Worldwide, the frequency of HCC onset, irrespective of gender, has significantly reduced among the mid-age population ranging from 30–59 years, largely due to the successful execution of Hepatitis B Virus (HBV) vaccination programs [3]. However, the incidence and mortality of HCC continue to rise especially because of the obesity pandemic which gives rise to NAFLD, and globally HCC mortality is expected to increase another 41% by 2040 [4]. When diagnosed early and when the tumor is <5 cm in size, liver transplantation and surgical resection are the treatment option for HCC [5]. However, a majority of the patients present at advanced stages because the disease is mostly asymptomatic in early stages. Advanced HCC is treated with RFA, TACE, TKIs and immunotherapy, although these modalities do not provide significant extension in lifespan with development of therapy resistance and disease recurrence [6]. As the disease progresses over the years, so are new diverse avenues for treating HCC being discovered. Advanced therapies in the field of cancer, targeting specific genes, have shown remarkable outcomes in recent years. At present there are more than 1000 clinical trials recruiting patients demonstrating the dedication towards finding novel and effective approaches for treating HCC [7]. A plethora of immune-based and gene-based therapies have been proven to be remarkable in treating cancer indicating their potential in HCC as well. Figure 1 provides a graphical representation of the different modes of therapeutic approaches to treat HCC.

## 2. Gene Therapy Approaches for HCC

Throughout its 32 years of history, the phenomenon of human gene therapy has been responsible for a remarkable contribution to the world of medical sciences, and its continuous modification and diverse approaches have made it possible to conquer the real daunting challenges in treating cancer by transforming it from bench to bedside [8]. The gene therapy approach aims at altering the genetic material inside a target cell. Genetic mutations can lead to gain of function or loss of function for a particular gene and turns it into a causative object to initiate cancerous growth. It is here where the crucial role of gene therapy techniques become involved to alter this defective genetic material by delivering therapeutic genes or gene-inhibitory molecules, such as small interfering ribonucleic acids (siRNAs), with a specialized approach to mitigate this crisis. Data has shown that in the case of HCC, gene therapy techniques manifested exceptional findings in treating patients worldwide. Gene therapy strategies might be in vivo, in which a gene product is delivered directly to a patient’s body using a viral or non-viral delivery system via intravenous, intra-arterial, intra-tumoral, intra-portal, intra-splenic or intra-biliary injection, or it might be ex vivo in which cells are genetically manipulated outside of the body and then injected back into the patients [9,10]. We will first discuss strategies of in vivo gene therapy with a description of different modalities of delivery systems.

### 2.1. Non-Viral Vector Mediated Gene Delivery

For gene therapy, a delivery system is required for introduction of the gene product to the target cell, and virus-based vectors serve as an efficient delivery system. Even though viral vector application in gene therapy has not been reported to cause major harm to the patients, its application is still a matter of controversial concern because of the stigma attached to viruses as well as a few isolated incidences of adverse effects. Non-viral gene therapy has been extensively studied and captivated sufficient attention from researchers as an effective strategy to treat HCC. A vector ought to be a responsible element to carry and precisely deliver the genetic material to its targeted destination without neglecting the concern for safety and efficacy. Some of the prime aspects of using non-viral vectors, such as efficiency of expressing genes with minimal immunogenicity and toxicity, manufacturing feasibility and cost effectiveness, render an added advantage to its wide acceptability as compared to viral vectors [11]. The most commonly used non-viral vectors are nanoparticles (NP), which are composed of polymers, lipids, peptides, inorganic particles, or a combination of these vectors. A number of non-viral vector-based approaches for HCC are currently under clinical trial, such as NCT02716012 and NCT04682847.

#### 2.1.1. Biodegradable and Non-Biodegradable Polymer Mediated Gene Delivery

Chitosan is a biodegradable linear polysaccharide of natural origin found mainly in the shells of shrimp and other crustaceans upon partial deacetylation [12]. The high polycationic properties, biocompatibility, low toxicity and efficient penetration properties made this nanoparticle and its derivatives a novel gene delivery system in HCC [13]. Chitosan-based nanoparticles loaded with ^125^I-labeled 5-Iodo-2′-Deoxyuridine showed significant accumulation of the nanoparticle in HCC cell line HepG2 compared to normal liver cells HL-7702. Internal irradiation caused by the drug caused lower DNA repair in HepG2 cells compared to HL-7702 cells and finally induced apoptotic cell death [14]. Doxorubicin, a widely used chemotherapeutic agent, is known to cause off-target cardiotoxicity. Chitosan nanoparticles loaded with ginger extract (GE) and doxorubicin was demonstrated to be highly effective against HCC, both in vitro and in vivo in a diethylnitrosamine (DEN)-induced HCC model, with a marked reduction in tumor cell viability and protection from cardiotoxicity [15]. A modified chitosan-based hybrid nanoparticle designed by aminoalkylation, MixNCH, was used to deliver siRNA for a potent oncogene midkine. Aminoalkylation was introduced with the notion that it will enhance the stability of the negatively charged siRNA-midkine to form a complex with modified chitosan and the efficiency of this strategy was demonstrated in HepG2 cells in vitro [16]. Galactose functions as a ligand for asialoglycoprotein receptors (ASGPR) which are specifically expressed in hepatocytes, thereby ensuring hepatocyte-specific delivery of the gene product. A galactosylated chitosan-polyethylene glycol (GCP) nanoparticle was used for the delivery of siRNA to the oncogene polo-like kinase 1 (*PLK1*) in a mouse xenograft model using HepG2 cells. Results from this in-vivo study revealed significant tumor regression accompanied by the upregulation of the pro-apoptotic molecules *p53*, *Bax* and *p21* and downregulation of anti-apoptotic molecule *Bcl-2* [17]. Triptolide (TP), a natural terpenoid, is a potent anti-cancer agent, including HCC. However, high toxicity, low water solubility and unknown therapeutic targets limit its clinical application. Galactosylated-chitosan-TP-nanoparticles (GC-TP-NP) facilitated high accumulation of TP in xenografts of SMMC-7721 HCC cells in vivo with a substantial reduction in tumor size and minimal systemic toxicity [18]. Similarly, GCP carrying gemcitabine showed significant anti-tumor efficacy against DEN-induced HCC in rats while depleting systemic toxicity [19]. A glycoprotein, asialofetuin, has high affinity for ASGPR, and chitosan-PLGA nanoparticles linked with asialofetuin was developed to deliver the anthracycline drug epirubicin (EPI-NPs). EPI-NPs, in combination with vitamin E derivative tocotrienols, displayed reduction in angiogenesis and an increase in p53-mediated apoptosis in a DEN-induced HCC mouse model with protection from cardiotoxicity [20]. A gene delivery construct with a modified GCP coupled to magnetic iron oxide revealed the efficient delivery of the potent tumor suppressor gene Ras association domain family member 1 (*RASSF1A*) precisely into HepG2 cells [21]. Orthotopic xenografts of HepG2 cells were established in nude mice which were treated with *RASSF1A*-carrying NP and mitomycin, and an external magnetic field was applied to the tumor area resulting in marked reduction in tumor with the induction of apoptosis.

Folate receptors are known to be present on the cell surface in many tumors including HCC. A chitosan nanoparticle (CNP) conjugated with folate (FA-CS-NP) was loaded with mouse interferon-γ-inducible-protein-10 (*IP-10*) for the purpose of immunotherapy. H22 tumor bearing mice, treated with FA-CS-NP, demonstrated inhibition of tumor growth with prolongation of survival time, which was accompanied by an induction of anti-tumor immune response [22]. Another folate-conjugated nano polymer, folate-PEI600-cyclodextrin (H1), was used to deliver the pro-apoptotic molecule tBid under the α-fetoprotein (*AFP*) promoter with the premise that since HCC cells express AFP, tBid will be expressed only in AFP-producing HCC cells. This strategy was shown to be efficacious in Hep3B and SK-Hep-1 xenograft models in nude mice with significant attenuation in tumor growth by increasing apoptosis and low toxicity [23].

Amylose, a natural biodegradable, biocompatible and non-toxic linear polysaccharide, is a promising carrier for gene therapy. A construct combining folate-functionalized, superparamagnetic iron oxide (SPIO)-loaded cationic amylose nanoparticles was generated for specifically targeting *survivin*, a potent oncogene in several cancers, including HCC, by siRNA. This strategy showed efficient silencing of *survivin* in HepG2 cells in vitro with the induction of apoptosis [24]. An added advantage of SPIO is visualization of the delivery process by MRI thereby serving as a theragnostic agent. A similar study conducted with SPIO loaded with siRNA radiolabeled by ^131^I to specifically target human vascular endothelial growth factor (*VEGF*), an oncogene playing a pivotal role in tumor angiogenesis and cancer progression, inhibited HepG2 xenografts in nude mice exposed to an external magnetic field with the concomitant downregulation of VEGF [25]. Another iron-oxide core coated with chitosan-polyethylene glycol-polyethyleneimine (PEG-PEA) co-polymer was conjugated with a monoclonal antibody for human glypican-3 (*GPC3*), highly expressed in HCC, thereby ensuring HCC-targeted delivery. Rat HCC cell line RH7777 was engineered to express luciferase and human GPC3 and orthotopic xenografts of these cells were treated with the nanoparticle delivering luciferase siRNA (NP-siRNA-GPC3AB). NP-siRNA-GPC3AB specifically bound to tumor and inhibited luciferase expression establishing proof-of-principle of the strategy [26].

An efficient non-biodegradable polymer is polyamidoamine (PAMAM), an amine terminated cationic dendrimer which is frequently used as a gene delivery system. Because of its high-density cationic charge, it confers an electrostatic interaction with different nucleic acids. Apoptin, a tumor-specific apoptosis-inducing protein, was loaded onto ornithine-conjugated PAMAM, and showed better transfection efficiency and intracellular uptake, which lead to apoptosis in HepG2 cells compared to PAMAM dendrimer alone [27]. The oncogene Astrocyte elevated gene-1 (*AEG-1*) is highly expressed in HCC and a galactose-conjugated PAMAM-PEG NP (PAMAM-PEG-Gal) delivering AEG-1 siRNA, in combination with all-trans retinoic acid (ATRA), markedly inhibited growth of orthotopic xenografts of QGY-7703 human HCC cells [28]. AEG-1 overexpression leads to NAFLD, a precursor of HCC, and PAMAM-PEG-Gal delivered AEG-1 siRNA protected mice from developing high fat diet (HFD)-induced NAFLD, further establishing the utility of this approach [29]. PAMAM dendrimer-delivered podophyllotoxin reduced inflammation, fibrosis and histological changes in the liver in DEN-treated mice, although the treatment was not continued long enough to measure the effect on tumor burden [30]. Doxorubicin delivered via N-acetylgalactosamine-conjugated PAMAM inhibited HepG2 xenograft in nude mice while providing protection from cardiotoxicity [31].

A newly developed biodegradable polymer poly-beta-amino-ester (PBAE) NP was loaded with secretable tumor necrosis factor alpha-related apoptosis-inducing ligand (sTRAIL) complementary deoxyribonucleic acid (cDNA), and in combination with histone deacetylase inhibitor (HDAC) inhibitors significantly inhibited growth of HepG2 xenografts. Interestingly, in vitro this approach induced apoptosis not only in transfected cells but also in non-transfected cells, suggesting a potential bystander effect [32].

#### 2.1.2. Lipid Based Non-Viral Gene Delivery

Both lipid and lipid-derived non-viral vectors have been explored as avenues for therapeutic gene transfer in HCC. Depending on the electronic charges, they are further categorized into cationic, neutral and anionic lipids [33]. Cationic lipid nanoparticles (LNP) interact better with nucleic acids due to its negative charge, and neutral and anionic lipids are suitable for drug delivery [34,35].

A lipid nanoparticle (LNP) was formulated by making a cocktail of cationic lipid (disteroylphosphatidyl choline), cholesterol, 1,2-dimyristoyl-sn-glycerol and methoxy-polyethylene-glycol and mixed with si-RNA for integrin β1 (*ITGB1*). This approach significantly knocked down *Itgb1* in mouse livers and inhibited spontaneous HCC in mice generated by the hydrodynamic injection of human MET and ΔN90-β-catenin plasmids along with sleeping beauty transposase [36]. LNPs are well known for their interaction with serum proteins, which can potentially direct LNPs to specific cell types. siRNA-loaded LNPs absorb apolipoprotein E (ApoE) on their surface, leading to binding with the low-density-lipoprotein-receptor (LDLR), thereby facilitating uptake by hepatocytes and HCC cells. This LDLR-mediated delivery mechanism has led to FDA approval of hepatocyte-targeted delivery of siRNA for an inherited disease [37]. Using a similar principle, LNP was generated to deliver siRNA for Jun N-terminal kinase-2 (Jnk2), which ameliorated hepatitis, fibrosis and the initiation of HCC in a spontaneous mouse model [38]. Defective Hippo/YAP signaling results in tissue overgrowth and development of HCC. LNP mediated YAP-siRNA successfully inactivated YAP, restored hepatocyte differentiation and lead to marked tumor regression in a genetically engineered mouse model of HCC [39].

LNP delivering interleukin-12 (IL-12) was evaluated for immune therapy in a MYC-driven HCC model. IL-12-LNP significantly reduced the tumor burden, had no effect on MYC levels, and elicited the pronounced infiltration of CD44^+^ CD3^+^ CD4^+^ T helper cells with increased production of IFN-γ, suggesting induction of an effective anti-tumor immune response [40].

Lipoplex (LPX) are a modified version of a lipid derived gene delivery system where a complex is formed due to the electrostatic interaction between a positively charged head group of cationic lipids and the negatively charged phosphate backbone of genomic material. A study was performed with LPX encoding the large non-structural protein 1 (NS1) of rat parvovirus (H-1PV) which induced multimodal cell death only in transformed cells. LPX-NS1 induced cell death in multiple HCC cell lines and inhibited the growth of Hep3B xenografts [41]. A novel and efficient non-viral gene carrier is pegylated immune-lipopolyplexes (PILP), a ternary complex formed with anionic liposomes, cationic polymer polyethyleneimine (PEI) and streptavidin-monoclonal antibody (Mab), the proof-of-principle of which was tested for plasmid DNA. The plasmid DNA is compacted by PEI, therefore it helps DNA entry into the nucleus, and lipopolyplexes account for minimal interaction with blood components and Mab promotes receptor-mediated endocytosis specifically to tumor cells. Although this study did not include any therapeutic experiment, it demonstrated efficient delivery of EGFP and luciferase only in H22 tumors in syngeneic mice [42].

Lipid/calcium/phosphate (LCP) NP conjugated with a galactose derivative was used to deliver VEGF siRNA and demonstrated tumor regression and anti-angiogenesis in a mouse orthotopic HCC model [43]. NP LN-DP1, consisting of 2-dioleyloxy-*N*,*N*-dimethyl-3-aminopropane (DODMA), egg phosphatidylcholine, cholesterol and cholesterol-polyethylene glycol was used to transfer miR-122, a liver specific tumor suppressor micro-RNA (miRNA) highly down regulated in HCC. Intratumoral injection of miR-122 mimic showed successful uptake of LN-DP1 by HCC cells, thereby reducing growth of SK-HEP-1 xenografts in nude mice [44]. Another study was conducted with this lipid complex by additional modification introduced by adding protamine to LCP (LCPP) for targeted delivery of TRAIL plasmid DNA (pDNA). Mice were treated with carbon tetrachloride (CCl_4_) to induce liver fibrosis following which mouse HCC cells HCA-1 were implanted in the liver and the mice were treated with a combination of LCPP-TRAIL and sorafenib, demonstrating the HCC regression amelioration of fibrosis [45].

LNP-siRNA-based therapy has moved from preclinical models to the clinical arena. Tabernero et al. describes the first trial using LNP to deliver siRNA for the VEGF and kinesin spindle protein (KSP), which resulted in the complete regression of liver metastases of endometrial cancer [46]. Although this study did not check the effect on HCC, the finding that the approach was able to induce target downregulation in liver documents its potential for HCC therapy as well.

#### 2.1.3. Peptide-Based Gene Delivery

Zein is an FDA approved maize protein that belongs to a class of prolamine. Half of the amino acid residues are hydrophobic in this peptide. Zein was PEGylated to form NP and further chemical modifications were carried out to increase biocompatibility and stability to use it as a photosensitizer in photodynamic therapy using HepG2 cells [47]. Two potent apoptotic genes, namely TRAIL and phosphatase and tensin homolog (*PTEN*), were loaded onto a Zein nanoparticle (ZNP) by a phase separation technique to check anti-tumor efficacy in HepG2 cells in vitro and in DEN-treated rats in vivo. This approach inhibited the proliferation of HepG2 cells and induced p53 and downregulated VEGF and matrix metalloproteinase-2 (MMP-2) in rats [48]. However, the study did not check the effect on in vivo tumor burden.

### 2.2. Viral Vector Mediated Gene Therapy Techniques

Even though non-viral delivery systems are showing promise in preclinical studies, there are very few clinical trials using this strategy in HCC. On the contrary, a virus-based gene therapy approach is being tested in multiple clinical trials because of target specificity and high transgene expression. Additionally, even though the virus-based gene therapy technique has its inherent risk and requires the ensuring of safety, lack of toxicity and efficacy in clinical trials, over the years numerous studies in multiple disease indications have proven that virus-based approaches are safe and efficacious. Viral vectors are derived from recombinant viruses including adenovirus (AD) and adeno associated virus (AAV), retrovirus (RV), lentivirus (LV), vaccinia virus (VACV), and herpes simplex virus (HSV). Most of the ongoing clinical trials for HCC are combinatorial studies of recombinant viral vectors with RFA, TACE, and hepatic artery infusion chemotherapy (HAIC), showing therapy response in HCC patients. Because of a large body of literature describing preclinical studies using viral vectors for HCC treatment, here we focus only on those approaches that have moved into the clinical arena. Table 1 and Table 2 summarize clinical trials using viral vector mediated gene therapy for HCC treatment.

#### 2.2.1. Adenovirus (AD) and Adeno Associated Virus (AAV)

Both of this double stranded DNA virus is used extensively as a gene delivery system because of their ability to infect both dividing and quiescent cells [49]. AD is the most common gene delivery approach in cancer gene therapy because it can infect various cell types, accommodate large DNA segments which can be expressed at high levels, and the technology to create high-titered clinical grade AD is already established. Another advantage of AD is that it persists in episomal form without integrating into the host genome, thereby preventing insertional mutagenesis and genomic irregularities. Human serotype 5 AD is most commonly used for gene therapy, and E1 and E3 genes, necessary for replication and evasion of immune response, respectively, are deleted to make the recombinant virus innocuous and safe for delivery [50]. TP53 is mutated in ~40–50% HCC cases and TP53 supplementation via AD is being evaluated in advanced HCC patients in clinical trials NCT02561546 (patients with diabetes) and NCT02509169 in combination with trans arterial embolization (TAE), in NCT02418988 in combination with TACE, and in NCT03544723 in combination with anti-programmed death 1 (anti-PD-1) or anti-programmed death-ligand 1 (anti-PD-L1) immunotherapy. NCT03544723 includes not only HCC but other solid tumors and lymphoma. A phase 2 clinical trial, NCT00300521, evaluating liver transplantation in combination with AD-mediated delivery of the suicide gene thymidine kinase (TK) has been completed, demonstrating overall survival of 54.8% at three years and a recurrence-free survival of 56.5%, both values being significantly higher than for liver transplantation alone. When the patients were stratified based on vascular invasion, overall and recurrence-free survival was 100% in patients with no vascular invasion. This strategy is currently being pursued in a Phase 3 study in NCT03313596. Another phase 2 study, NCT02202564, in unresectable HCC patients with >5 cm tumor without extrahepatic metastasis evaluating AD-TK along with the drug ganciclovir in combination with liver transplantation has been completed, although no result has yet been posted. A phase 1 study, NCT00844623, testing intratumoral injection of AD expressing TK of herpes simplex virus (HSV) followed by systemic ganciclovir in 10 HCC patients has been completed [51]. Each patient received up to three injections at a dose of 10^10^ to 2 × 10^12^ viral particles (vp) at 30 days interval. The treatment was well tolerated with flu-like symptoms an no dose-limiting toxicity. Sixty percent of patients showed stabilization of the injected tumor and two patients receiving the highest dose showed signs of intratumoral necrosis by positron emission tomography (PET), with one surviving up to 26 months. This study established the safety and partial efficacy of AD.TK therapy.

There are many strains of AAV that are used for gene therapy purposes, some of which persist in an episomal state, while some integrate into the genome. AAVs exhibit low pathogenicity and no cytotoxicity and thus serve as a suitable vector for gene therapy [49]. AAV serotype 8 (AAV8) is specifically suitable for gene delivery in the liver, exerting high affinity to hepatocytes and transducing 90–95% hepatocytes via intraportal injection in mice [52]. AAV8-mediated gene or miRNA delivery has been effective in delaying HCC progression in mouse models [53,54]. Although there are many AAV-mediated clinical trials in cancers and other diseases, and there are 2 FDA-approved AAV-based gene therapy products, Luxturna (voretigene neparvovec-rzyl) for retinal dystrophy and Zolgensma (onasemnogene abeparvovec-xioi) for spinal muscular atrophy, there are no ongoing clinical trials for AAV-mediated gene therapy in HCC [55]

#### 2.2.2. Lentivirus (LV)

LV is a single stranded RNA virus that belongs to the genus retrovirus. Most of the lentivirus vectors used in gene therapy are modified from Human Immunodeficiency Virus-1 (HIV-1) [56]. LV gets reverse transcribed and integrates into the host genome as double stranded DNA. Upon integration it uses host cell machineries and transcribes the transgene. LV can infect both dividing and non-dividing cells, thereby making it a suitable vector for gene therapy. Additionally, because of its ability to integrate, lentiviruses are used to deliver shRNA ensuring persistent knockdown of the target gene. In the clinic, LV is mainly used in CAR-T-based therapy, which will be discussed in a later section. Currently there are no clinical trials for HCC in which LV is used for in vivo gene therapy.

#### 2.2.3. Herpes Simplex Virus (HSV)

HSV is a neurotropic double-stranded DNA virus, and its highly infectious characteristics makes it an excellent vector in gene delivery approaches in treating cancers including HCC [57]. In clinical trials, NCT04336241, a genetically modified HSV-1 expressing anti-cytotoxic T-lymphocyte associated protein 4 (anti-CTLA-4) antibody, is being evaluated to directly destroy tumors and generate an anti-tumor immune response. This trial includes 36 patients with a variety of solid cancers, including gastrointestinal (GI) cancers.

#### 2.2.4. Vaccinia Virus (VACV)

VACV is comprised of linear double stranded DNA, belonging to the family of poxviridae. Modified vaccinia virus Ankara (MVA) is a highly attenuated strain of VACV, shown to be effective and safe for gene therapy and vaccination [58]. TG4023 is an MVA expressing cytosine deaminase and uracil phosphoribosyl transferase enzymes that transform the prodrug flucytosine (5-FC) into cytotoxic 5-fluorouracil (5-FU) and 5-fluorouridine-5′-monophosphate, respectively, and its safety and efficacy in primary or secondary hepatic tumors was tested in a clinical trial NCT00978107 [59]. It was shown that intratumoral injection of TG4023 was feasible and well tolerated, and the maximal tolerated dose (MTD) was defined as 4 × 10^8^ plaque forming units (pfu). A therapeutic 5-FU concentration was achieved, and eight of 16 patients had stable disease.

### 2.3. Oncolytic Viruses

Oncolytic viruses are genetically modified viruses that demonstrate a promising ability to specifically replicate in and lyse infected cancer cells without affecting adjacent normal cells. Oncolytic viruses account for the elimination of tumor cells in two ways, they specifically infect cancer cells and undergo viral replication leading to cell lysis and induce cell-mediated tumor specific immunity [60]. Table 2 summarizes clinical trials using oncolytic viruses for HCC treatment.

#### 2.3.1. Oncolytic Ad

Upon AD infection, the tumor suppressor p53 in host cell functions as a checkpoint to stall the cell cycle at the S phase, which prevents viral replication. The E1B protein of AD inactivates host p53 to facilitate its own replication. Upon deletion of E1B, AD fails to replicate in normal cells having wild type p53. In ~40–50% cases of HCC, the p53 gene is mutated and inactivated, and as such an E1B-deleted AD will replicate in p53-mutated HCC cells (or any other cancer cells), resulting in replication-induced cytolysis [61]. This E1B-deleted recombinant human adenovirus type 5 is being evaluated for unresectable HCC in the clinical trial NCT01869088 in combination with TACE, in NCT03790059 in combination with RFA, and in NCT05113290 in combination with sorafenib.

SynOV1.1 is an oncolytic Ad with deletion of E1B and partial E3 genes and incorporation of E1A and human granulocyte macrophage colony-stimulating factor (hGM-CSF) genes under AFP promoter [62]. A Phase 1/2 study (NCT04612504) is currently ongoing to test the safety, tolerability and efficacy of SynOV1.1, either alone or in combination with the anti-PD-L1 antibody atezolizumab in 45 advanced HCC patients.

#### 2.3.2. Oncolytic VACV

Pexastimogene devacirepvec, (JX-594 or Pexa-Vec) is a VACV with disruption of the viral TK gene and a transgene for granulocyte macrophage colony-stimulating factor (GM-CSF). Cancer cells exhibit high cellular TK activity and activated epidermal growth factor receptor (EGFR) signaling which are required for VACV replication. As such, JX-594 selectively infects and replicates within tumor cells. Infection of tumor cells by JX-594 induces an adaptive immune response [63]. A phase I clinical trial (NCT00629759) was completed with JX-594 in patients with primary or metastatic liver cancer. MTD for intratumoral injection of JX-594 was 10^9^ pfu, and it was generally well-tolerated with direct hyper bilirubinemia as dose-limiting toxicity. Acceptable safety was observed in the patients in terms of JX-594 replication, GM-CSF expression and systemic dissemination. Among 14 patients completing the treatment, 10 could be evaluated radiographically, of which three showed partial response, six had stable disease and one had progressive disease [64]. This initial phase 1 study was followed by a randomized phase 2 study (NCT00554372) in 30 unresectable HCC patients demonstrating oncolytic and immunotherapy responses with survival duration of 14.1 months and 6.7 months on the high and low dose of the drug, respectively [65]. A phase 3 trial (NCT02562755) of Pexa-Vec in combination with sorafenib was completed in 2020 in 459 HCC patients, and initial results showed that the combination treatment is mostly well-tolerated, although the full result of the study is yet to be published. A phase IIb trial of Pexa-Vec (NCT01387555) in 129 HCC patients after sorafenib failure unraveled a tolerable safety profile but no improvement in overall survival as a second line therapy, suggesting that oncolytic virus treatment might be more efficacious in earlier stages of the disease [66]. A phase 1 trial (NCT03071094) of a Pexa-Vec and anti-PD-1 antibody Nivolumab combination was prematurely terminated.

Another genetically engineered oncolytic VACV, PF-07263689, in combination with the anti-PD-1 antibody sasanlimab entered a Phase 1 trial (NCT05061537) in late 2021 in 120 solid cancer patients including HCC.

#### 2.3.3. Other Oncolytic Viruses

M1 is a single-stranded RNA virus, isolated from a pool of mosquitoes in China, which has been shown to not cause any human or animal disease. Interestingly, M1 specifically infected different types of cancer cells, inducing replicative cell death without exerting detrimental effects to normal cells and IV injection of M1 significantly suppressed growth of Hep3B xenografts in nude mice [67]. M1 in combination with anti-PD-1 antibody and TKI apatinib is being evaluated in 10 HCC patients in a Phase 1 trial (NCT04665362).

VG161 is an oncolytic HSV-1 OV expressing IL-12, IL-15 with its receptor α unit, and PD-L1 antagonist (Fc-fused 14 amino acid peptide) that induces oncolysis and boosts T cells and the NK cell-mediated anti-tumor immune response [68]. A phase 2 single arm trial (NCT05223816) was initiated in February 2022 in 41 patients with HCC or intrahepatic cholangiocarcinoma to evaluate the efficacy, safety and tolerability of VG161. It should be noted that another oncolytic HSV-1 expressing GM-CSF, named IMLYGIC (Talimogene Laherparepvec), has been approved by the FDA for the treatment of melanoma [69].

### 2.4. Suicide Gene Therapy

Among the several cancer gene therapy approaches, suicide gene therapy involves a unique strategy of incorporating the selective transformation of a non-toxic compound into a cytotoxic drug within the cancer cells. The rationale behind integrating the suicide gene under the control of a tumor or cell-specific specific promoter is that it restricts the suicide inducing transgene expression precisely inside the tumor cell leading to apoptosis while minimizing the impact on normal healthy adjacent cells [70]. This method has been proven to be more efficient in treating solid tumors and chemo-resistant patients. Suicide gene therapy is also crucial for enhancing the efficacy of radiotherapy [71].

Suicide gene therapy is also known as Gene Directed Enzyme Prodrug Therapy (GDEPT). The most common genes that are introduced into tumor cells are mainly viral and bacterial genes. The two prime systems that has been used extensively in suicide gene therapy are the HSV thymidine kinase (HSV-TK) gene, which converts ganciclovir (GCV) to ganciclovir monophosphate [72], and the cytosine deaminase gene (CD) of Escherichia coli, which is responsible for the conversion of pro-drug 5-Fluorocytosine (5-FC) to 5-Fluorouracil (5-FU) [73]. In addition, purine nucleoside phosphorylase (PNP) is an E. Coli enzyme that converts the prodrug fludarabine phosphate (FP) to the active drug, 2-fluoroadenine [74].

An in-vitro study was performed using HSV/TK under the control of a survivin promoter, showing selective killing after GCV treatment in HepG2 cells but not in LO2 normal human liver cells [75]. In an immunocompetent model in which MM45T.Li mouse HCC cells were subcutaneously (s.c.) injected into syngeneic mice, ADs delivering HSV/TK and a chimera of monocyte chemoattractant protein-1 (MCP-1) and the membrane-spanning domain of CX3CL1 (fractalkine), displayed high antitumor efficacy upon GCV treatment. It was concluded that MCP-1 promoted the recruitment and activation of macrophages and T cells, thus augmenting the anti-tumor effect upon apoptosis induction by HSV-TK/GCV [76]. Adenovirus, encoding the HSV-TK gene driven by hTERT-targeting *trans*-splicing ribozyme under the control of liver-specific phosphoenolpyruvate kinase (*PEPCK*) promoter with an ApoE enhancer placed in the distal region of the HSV-TK expression cassette, showed significant anti-tumor efficacy in a multifocal HCC model with splenic subcapsular inoculation of Hep3B cells in nude mice without damaging normal hepatocytes. Noninvasive PET imaging was able to check HSV-TK expression in the tumor as well as tumor growth [77]. HSV-1 viral vector harboring cytosine deaminase (CD) induced killing in both HCC and non-HCC cells in vitro and inhibited xenografts of primary HCC 26-1004 cells in nude mice upon treatment with 5-FC [78]. In another study, double suicide gene system TK and CD was delivered via cationic microbubbles decorated with αVβ3 integrin antibody to specifically target HepG2 cells. This approach inhibited HepG2 xenografts in nude mice by the induction of apoptosis upon treatment with GCV and 5-FC [79].

An ultrasonic nanobubble-mediated delivery of PNP/fludarabine suicide gene system induced cytotoxic effects on HepG2 and SMC7721 cells upon exposure to ultrasound and exerted a bystander effect [80]. Suicide gene therapy has moved from preclinical studies to clinical trials (NCT00844623), strengthening potential efficacy of this approach in HCC treatment. However, there still exist a few loopholes, such as the lack of an efficient delivery process, the conversion rate of pro-drug, short span and low-profile expression of the transgene and bystander effect, which need to be addressed efficiently to translate this gene therapy approach from bench to bedside.

### 2.5. Clustered Regularly Interspaced Short Palindromic Repeats and CRISPR-Associated Protein 9 (CRISPR/Cas9)

The CRISPR/Cas9 system is an excellent genome editing tool for interpreting the molecular fundamentals of drug resistance and refining the clinical outcomes. The system consists of a guide RNA which directs the system to a target sequence and the Cas9 nuclease which cleaves the double stranded DNA at that specific location [81]. It was first identified in bacteria regulating adaptive immune response as a defensive mechanism against phage infection [82]. The wide range of CRISPR/Cas9 application leads to the opening of diverse horizons in medical science. In case of gene therapy in HCC patients, a CRISPR/Cas9 system has made a remarkable contribution in finding potential diagnostic and therapeutic targets. Genome-wide CRISPR/Cas9 screening identified that Kelch-like ECH associated protein 1 (*KEAP1*) mediates susceptibility to TKIs, such as sorafenib, lenvatinib, and regorafenib in HuH-7 HCC cells. KEAP1 inactivation led to activation of the antioxidant Nrf2 transcription factor, leading to decreased reactive oxidant species (ROS) levels that mediated resistance to TKIs [83]. A similar screening strategy identified neurofibromin 1 (NF1) and dual specificity phosphatase 9 (*DUSP9*) as drivers for lenvatinib resistance in HuH-7 cells. It was shown that loss of NF1 reactivated the PI3K/AKT and MAPK/ERK pathways, while DUSP9 loss activated MAPK/ERK pathways resulting in lenvatinib resistance. These two studies demonstrate utilization of CRISPR/Cas9 screening to unravel mechanism of drug resistance for the development subsequent targeted therapy, which may include gene therapy approaches. A targeted delivery system was created by the adsorption of aptamer against epithelial cell adhesion molecule (*EPCAM*), a marker of HCC stem cells, and PAMAM onto hollow mesoporous silica nanoparticles (HMSN) for the co-delivery of sorafenib and CRISPR/Cas9 system targeting EGFR. This approach successfully inhibited 85% of tumor growth in an HCC model using mouse H22 cells, with no damage to major organs [84]. Another HCC therapy in combination with sorafenib was generated by constructing charge-reversal nanocomplex, consisting of a negatively charged heparin core and positively charged ethanolamine (EA)-modified poly(glycidyl methacrylate) (PGEA) shell, termed Hep@PGEA, to deliver pCas9 and sgRNA targeting survivin [85]. In an orthotopic mouse model of BEL-7402 human HCC cells, this combination treatment showed a marked reduction in tumor growth compared to either agent alone without exerting any toxicity. Similar survivin-targeted strategy using lactose-derived branched-cationic biopolymer (LBP), with the rationale that lactose will bind to ASGPR providing targeted delivery, in BEL-7402 orthotopic model also showed marked tumor reduction without any toxic effect [86]. These studies demonstrate that specifically targeting survivin by CRISPR/Cas9 provides a safe and attractive strategy for HCC management with promising clinical translation. An in vitro study demonstrated that CRISPR/Cas9 mediated knock out of long non-coding RNA IncRNA-RP11-156p1.3 in HepG2 cells induced a significant decrease in cell viability showing its potential role in regulating HCC pathogenesis [87]. Sonodynamic therapy (SDT) is a well-known cancer treatment paradigm consisting of the synergistic interaction between ultrasound and chemical agents (sonosensitizers), and its effectiveness has been demonstrated in both in vitro and ex vivo studies. The principle of this therapy is to disrupt the cell functions by ultrasound, which kills cancer cells by expressing the production of ROS activated by sonosensitizers [88]. Nuclear factor, erythroid derived 2, like 2 (*NFE2L2*, also known as Nrf2) is activated during SDT, inhibiting SDT efficacy. Cationic liposomes were loaded with pCas9 and NFE2L2 sgRNA along with the sonosensitizer hematoporphyrin monomethyl ether (HMME), which profoundly inhibited HepG2 xenografts upon ultrasound administration in nude mice. The system was shown to exert no hepatorenal toxicity [89]. The CRISPR/Cas9 system is also effective as an ex-vivo gene therapy, for instance, the CRISPR/Cas9 approach is currently being evaluated in a clinical trial to knockout PD-1 receptor in autologous T-cells that are extracted from HCC patients undergoing TACE treatment (NCT04417764).

## 3. Immunotherapy Approaches for HCC Treatment

Liver is the largest internal organ which has a binary blood supply from the hepatic artery and portal vein. It has a unique role in promoting immune tolerance. Due to continuous exposure of normal gut flora through the portal vein, the liver needs to acquire suppressive immune activity to restrain unnecessary immune responses. Immune tolerance is mediated by immune suppressive cytokines, myeloid derived suppressor cells (MDSC), and regulatory T-cells (Treg) [90]. An immune tolerant environment promotes malignant hepatocytic growth that fails to be diagnosed in early stages. Immuno-suppressive and immuno-activating cells play a contrasting pivotal role in HCC. It has been shown that increased expression of CD4+/CD25+/forkhead/winged helix transcription factor (FoxP3)+ Tregs correlate with reduced effector CD8+ T cell infiltration in the tumors and poor survival in HCC patients [91]. Increased numbers of Tregs, MDSC and exhausted T-cells and increased levels of immunosuppressive cytokines were detected in advanced HCC patients compared to normal control [92].

Key molecules, modulating anti-tumor T cell responses, regulate immune checkpoints and are expressed by T cells, antigen-presenting cells, such as macrophages and dendritic cells, as well as tumor cells [93]. Programmed Cell Death 1 (PD-1/PDCD1), cytotoxic T-lymphocyte associated protein 4 (CTLA4), lymphocyte activating 3 (LAG3/CD223) and T cell immunoglobulin and mucin domain-containing protein 3 (TIM3/HAVCR2/CD366) serve as the principal immune check point receptors that inhibit T cell activity and maintain self-tolerance. Co-stimulatory molecules which augment T cell expansion include TNF receptor superfamily member 4 (TNFRSF4/OX40/CD134), glucocorticoid-induced TNF receptor (GITR/TNFRSF18) and CD28. Interaction between PD-1 with PD-1 ligand 1 (PD-L1/B7H1/PDCDL1/CD274), expressed by tumor cells, results in dephosphorylation of T cell activating kinases, causing T cell activation, and as such the inhibition of PD-1/PD-L1 restores function of effector CD8+ T cells [94]. On the other hand, the inhibition of CTLA4 potentiates the interaction of co-stimulatory molecule CD80/B7 with CD28 at the immune synapse of T cells and antigens presenting cells augmenting the activation of naïve CD4+ and CD8+ T cells [95]. The activated T cells, induced by PD-1 or CTLA4 blockade, can efficiently induce the killing of cancer cells, thereby establishing the principle of immune checkpoint inhibitor (ICI) therapy. Overexpression of PD-L1 is highly associated with overall poor prognosis, tumor stage and recurrence risk of HCC, thereby establishing the rationale of using ICI therapy with PD-1 inhibitors [96]. An immune classification of HCC identified an ‘immune’ class, accounting for ~25% of HCC, which is characterized by a high level of immune infiltration, increased PD-1/PD-L1 signaling and enrichment of transcriptional signatures that show response to ICI therapy in other cancers [97]. As such, it was hypothesized that this class of HCC patients will respond well to ICI therapy [98].

Nivolumab is a human IgG4 antibody against PD-1 which was the first immunotherapy approved for HCC by the FDA as a second line therapy, following sorafenib treatment, or as a first line treatment if patients are ineligible or intolerant to other first-line treatments, upon completion of the CheckMate 040 trial, in September 2017 [99,100]. Atezolizumab is a fully humanized, monoclonal IgG1 isotype antibody against PD-L1, and bevacizumab is a humanized antibody against VEGF. In a phase 1b GO30140 trial, atezolizumab and bevacizumab in combination showed efficacy with an overall response rate (ORR) of 27% (*n* = 60) [101]. This combination was subsequently studied in a phase three trial (IMbrave150) comparing its efficacy vs. sorafenib in treatment-naïve advanced HCC patients (*n* = 501) [102]. Compared to sorafenib, the combination demonstrated longer median overall survival (OS) (not reached vs. 13.2 months, HR 0.58, *p* = 0.0006) and progression free survival (PFS) (6.8 versus 4.3 months). The ORR for the combination and sorafenib was 33.3% vs. 13.3%, respectively. The most adverse events (AE) in the combination arm included hypertension (15.2%), increased aspartate aminotransferase (7%), thrombocytopenia (3.3%), and proteinuria (3%). On May 29, 2020, the US FDA approved atezolizumab and bevacizumab in combination as a first-line therapy for treating advanced unresectable HCC patients [103]. Following the success of this approach, a plethora of clinical trials are being performed to investigate the different combinations of immunotherapy in HCC patients, which is summarized in Table 3 and Table 4. ORIENT-32 is a randomized, open-label phase 2/3 study done in China comparing the efficacy of sintilimab (a PD-1 inhibitor) plus IBI305, a bevacizumab mimic, versus sorafenib as a first-line treatment for unresectable HBV-HCC [104]. The combination treatment group showed significantly longer median PFS (4.6 months) compared to sorafenib (2.8 months), and median OS (10.4 months for sorafenib, not reached in the combination group). In a phase 1b study (NCT03006926) in 104 HCC patients, lenvatinib plus pembrolizumab combinations showed an ORR of 36% and median OS and PFS of 22 and 8.6 months, respectively [105]. AE of grade 3 or more was observed in 71% patients, most common being hypertension (17%), with three patients dying during treatment. One of the consequences of ICI treatment is adverse effects, including the development of autoimmunity because of augmented immune activity [106]. Nevertheless, combination immunotherapy seems to become the treatment of choice for all HCC patients, and the completion of the ongoing Phase 3 trials will provide further rationale in this context.

## 4. Chimeric Antigen Receptor (CAR)-T Cell Therapy

CAR-T therapy is an ex vivo gene therapy aimed at reprogramming the immune system for treating cancer. Cancer cells expressing cancer cell-specific antigens and CARs are synthetically modified recombinant receptors that bind to specific antigens expressed on patients’ cancer cells. T-cells are isolated from patients’ blood, modified to express CARs via lentivirus vectors and then reintroduced back to the patients. These activated T cells recognize the antigen-expressing cancer cells and kill them [107]. Antigen selection for CAR designing is a daunting challenge, as solid tumor antigens are also frequently expressed on the non-cancer cell surface, hindering therapeutic efficacy [108]. There are a few common targets for CAR-T cell therapy in HCC, the most common being Glypican-3 (GPC3), a 70 kDa heparan sulfate proteoglycan, expressed in approximately 75% of HCC patients but not by normal hepatocytes. Studies have shown the promising efficacy of GPC3-CAR-T in suppressing tumor growth by patient derived xenografts (PDXs) of HCC [109]. As such, GPC3-CAR-T is being evaluated in multiple Phase 1 clinical trials (Table 5). GPC3-CAR-T expressing IL-15 is being evaluated in clinical trial NCT05103631 with the rationale that IL-15 boosts the efficacy and viability of CAR-T cells. Additional targets include AFP, EPCAM, MUC1 (Mucin 1) and CD147 (Cluster of Differentiation 147), which are being evaluated in clinical trials [110]. All of these clinical trials are still in the recruitment phase, and the efficacy is yet to be determined. One hindrance in the effective use of CAR-T therapy is toxicity, which includes cytokine release syndrome, target miss-effect and off-target effects [111]. A potential approach has been formulated to mitigate CAR-T cell toxicity by implementing “off switches” or a suicide gene strategy to selectively reduce CAR-T cells upon the occurrence of an adverse event by the help of a secondary agent [112]. One approach of constructing CAR-T cell involves expressing chemokine receptors on CAR-T cells that complement and respond to tumor-derived chemokines [113]. CAR-T cell therapy is effective in liquid cancers, and in 2017, the FDA has approved Tisagenlecleucel (trade name: Kymriah) for acute lymphoblastic leukemia and diffuse large B-cell lymphoma (DLBCL), and Axicabtagen ciloleucel (trade name: Yescarta) for DLBCL and Non-Hodgkin’s lymphoma [114,115]. A number of BCMA-targeted CAR-T cell therapies have also been FDA-approved for multiple myeloma [116]. Thus, with the completion of the ongoing clinical trials, CAR-T therapy for HCC might also emerge as a first-line treatment strategy.

## 5. Conclusions and Future Direction

HCC is a disease of chronic inflammation due to a variety of causes. The chronic inflammatory process causes hepatocyte injury, setting forth a process of hepatocyte proliferation and apoptosis, coupled with the activation of stellate cells inducing fibrosis. Extensive fibrosis leads to cirrhosis of the liver, which severely compromises liver functions and in this background mutation develops in hepatocytes causing HCC. Liver is the primary metabolic organ of the body. The destruction of the normal liver by the cirrhotic process adversely affects the drug metabolizing capacity of the liver, therefore one of the major reasons for treatment failure is drug-induced toxicity which significantly reduces patients’ compliance in taking chemotherapeutic drugs. In this context, it is important to do research and develop modalities of treatment that are targeted, safe and non-toxic and efficacious in providing significant survival benefits to the HCC patients. Immunotherapy (either mono or combinational) has shown higher potentials after combining with adjuvants, such as, curative resection, surgical ablation or TACE at phase III trials. Combinatorial immuno-therapeutic approaches (atezolizumab and bevacizumab) demonstrate positive outcomes at an advanced stage of the disease and are being recommended for administration even at early stages. Liver displays high target organ delivery of a payload after IV administration and direct delivery to liver can be achieved by placing a catheter in the portal vein of the hepatic artery. This advantage is being exploited in clinical trials using different shades of gene therapy techniques, such as suicide gene therapy, viral and non-viral vector mediated gene therapies. However, there are still issues of transfection efficiency, intracellular interference, target specificity and toxicity and safety concerns regarding viral vector-based gene therapy, which are being continuously worked to develop safer and more targeted delivery approaches. Non-viral vector mediated gene delivery system is low in their cytotoxicity and immunogenicity, and LNP-mediated delivery approaches are showing promise in clinical trials. In recent years, the introduction of a CAR-T cell and CRISPR/Cas9 gene delivery strategy has created high hopes for effective treatment of HCC, and these approaches are now under phase 3 trials. It is important to understand the molecular pathogenesis in-depth and in a comprehensive way, and to stratify patients based on molecular and immunological classification to identify the best possible treatment strategy. The advancement of immunological, biological and targeted therapy approaches will help establish personalized medicine for HCC patients providing a significant improvement of quality of life and meaningful extension of overall and disease-free survival.

## Figures and Tables

**Table 1:** Data from https://www.clinicaltrials.gov/ accessed on 10 May 2022 showing clinical trials viral vector mediated gene therapy for the treatment of advanced hepatocellular carcinoma.

Study Identifier	Intervention	Phase	Study Status	Entry Routes	Adjuvant	Patients Enrolled
NCT02561546	rAd-p53	Phase 2	Not yet recruiting	Arterial infusion	TAE	Diabetes concurrent with HCC
NCT02509169	rAd-p53	Phase 2	Recruiting	Arterial infusion	TAE	Advanced HCC
NCT02418988	rAd-p53	Phase 2	Recruiting	Arterial infusion	TACE	Advanced HCC
NCT03544723	Ad-p53	Phase 2	Recruiting	Intratumoral injection	anti-PD-1 (ICI)	Solid tumor of HCC
NCT00300521	ADV-Tk	Phase 2	Completed	N/A	Liver transplant	Advanced HCC
NCT03313596	ADV-Tk	Phase 3	Recruiting	Peritoneum tissue around the liver	Liver transplant	Advanced HCC
NCT02202564	ADV-Tk	Phase 2	Completed	Peritoneum injection	Liver transplant	Advanced HCC
NCT00844623	ADV-HSV-TK	Phase 1	Completed	Intratumoral injection	---	HCC
NCT00669136	AFP-AdV	Phase 1	Terminated	Intramuscular	---	HCC
NCT04336241	HSV-1-RP2	Phase 1	Recruiting	Intratumoral injection	---	HCC
NCT00978107	MVA-FCU1	Phase 1	Completed	Intratumoral injection	---	HCC

**Table 2:** Data from https://www.clinicaltrials.gov/ accessed on 10 May 2022 showing clinical trials of oncolytic virus mediated gene delivery for the treatment of advanced hepatocellular carcinoma.

Study Identifier	Intervention	Adjuvant	Phase	Study Status	Entry Routes	Patients Enrolled
NCT04612504	SynOV1.1	atezolizumab	Phase 1	Not yet recruiting	Intratumoral injection	Patient with AFP positive advanced HCC
NCT01869088	rhAdV type-5	Phase 3	Active, not recruiting	Arterial infusion	TACE	Unresectable HCC
NCT03790059	rhAdV type-5	N/A	Recruiting	Intraoperative injection	RFA	HCC
NCT05113290	rhAdV type-5	Phase 4	Active, not recruiting	Intratumoral injection	Sorafenib	Advanced HCC
NCT00629759	JX-594 (Pexa-Vec)	---	Phase 1	completed	Transdermal injection	patients with advanced HCC
NCT00554372	JX-594 (Pexa-Vec)	---	Phase 2	Completed	Intratumoral injection	Unresectable primary HCC
NCT02562755	Pexa-Vec (JX-594)	Sorafenib	Phase 3	Completed	Intratumoral injection	HCC patients
NCT01387555	JX-594 (Pexa-Vec)	---	Phase 2	Completed	N/A	patients with advanced HCC
NCT03071094	Pexa-Vec (JX-594)	Nivolumab	Phase 1	Terminated	Intratumoral injection	patients with advanced HCC
NCT05061537	PF-07263689	Sasanlimab	Phase 1	Recruiting	Intravenous infusion	metastatic solid tumors with HCC
NCT04665362	M1 (M1-c6v1)	Apatinib	Phase 1	Not yet recruiting	Intravenous infusion	patients with advanced HCC
NCT05223816	VG161	---	Phase 2	Not yet recruiting	Intratumoral injection	HCC patients

**Table 3:** Data from https://www.clinicaltrials.gov/ accessed on 10 May 2022 showing clinical trials of immunotherapy (immune checkpoint inhibitor monotherapy) for the treatment of advanced hepatocellular carcinoma.

Study Identifier	Intervention	Target	Phase	Study Status	Entry Routes	Patients Enrolled (Enrolment Target)
NCT04294498	Durvalumab	PD-1	Phase 2	Recruiting	Intravenous infusion	HCC patients with active chronic HBV infection (43)
NCT04268888 (TACE-3)	Nivolumab	PD-1	Phase 2/3	Recruiting	Intravenous infusion	Intermediate stage HCC all receiving TACE/TAE and half receiving nivolumab (522)
NCT02576509	Nivolumab	PD-1	Phase 3	Active, not recruiting	Intravenous infusion	First line treatment with advanced HCC patient; compares efficacy versus sorafenib treatment (743: 371 nivolumab; 372 sorafenib)
NCT03412773 (RATIONALE-301)	Tislelizumab	PD-1	Phase 3	Active, not recruiting	Intravenous infusion	Patients with unresectable HCC; compares efficacy versus sorafenib treatment (674)
NCT0270241MK-3475-224/KEYNOTE-224)	Pembrolizumab	PD-1	Phase 2	Active, Not recruiting	Intravenous infusion	Advanced HCC patients (156)
NCT03062358 (MK-3475-394/KEYNOTE-394)	Pembrolizumab	PD-1	Phase 3	Active, not recruiting	Intravenous infusion	Advanced HCC patients; compared to placebo given with best supportive care (454)
NCT03867084 (KEYNOTE-937)	Pembrolizumab	PD-1	Phase 3	Recruiting	Intravenous infusion	As adjuvant therapy in HCC patients with complete radiological response after surgical resection or local ablation; compared to placebo (950)
NCT03383458 (CheckMate 9DX)	Nivolumab	PD-1	Phase 3	Active, Not recruiting	Intravenous infusion	As adjuvant therapy in HCC patients with complete surgical resection or complete response after local ablation; compared to placebo (545)
NCT03859128 (JUPITER 04)	Toripalimab	PD-1	Phase 2/3	Active, Not recruiting	Intravenous infusion	As adjuvant therapy in HCC patients with complete surgical resection; compared to placebo (402)

**Table 4:** Data from https://www.clinicaltrials.gov/ accessed on 10 May 2022 showing clinical trials of combinational immunotherapy (immune checkpoint inhibitor) for the treatment of advanced hepatocellular carcinoma.

Study Identifier	Intervention	Target	Phase	Study Status	Entry Routes	Patients Enrolled (Enrolment Target)
NCT04712643	Atezolizumab + Bevacizumab	PD-L1, VEGF	Phase 3	Recruiting	Intravenous infusion	Untreated HCC patients; compares with TACE alone or TACE with combination therapy (342)
NCT04803994	Atezolizumab + Bevacizumab	PD-L1, VEGF	Phase 3	Recruiting	Intravenous infusion	Intermediate stage HCC patients; compares with TACE alone or TACE with combination therapy (434)
NCT04102098 (IMbrave050)	Atezolizumab + Bevacizumab	PD-L1, VEGF	Phase 3	Active, not recruiting	Intravenous infusion	Patients with completely resected or ablated HCC who are at high risk of disease recurrence; compares with active surveillance (668)
NCT03434379 (IMbrave150)	Atezolizumab + Bevacizumab	PD-L1, VEGF	Phase 3	Active, not recruiting	Intravenous infusion	Untreated locally advanced HCC; compares efficacy versus sorafenib treatment (336 immunotherapy; 165 sorafenib)
NCT04770896 (IMbrave251)	Atezolizumab + Lenvatinib or Sorafenib	PD-L1, TKI	Phase 3	Active, not recruiting	Intravenous infusion	Unresectable HCC; compares efficacy or combination versus lenvatinib or sorafenib (554)
NCT04039607 (CheckMate 9DW)	Nivolumab + Ipilimumab	PD-1, CTLA-4	Phase 3	Active, not recruiting	Intravenous infusion	Advanced HCC; compares efficacy versus sorafenib or lenvatinib treatment (728)
NCT03006926	Lenvatinib + Pembrolizumab	TKI, PD-1	Phase 1	Active, not recruiting	Capsule & IV respectively	Patients with HCC (104)
NCT05027425	Durvalumab + Tremelimumab	PD-1, CTLA-4	Phase 2	Recruiting	Intravenous infusion	Patient listed for liver transplant (30)
NCT04340193 (CheckMate 74W)	Nivolumab + Ipilimumab	PD-1, CTLA-4	Phase 3	Active, not recruiting	Intravenous infusion	Patients with intermediate HCC (40)
NCT03510871	Nivolumab + Ipilimumab	PD-1, CTLA-4	Phase 2	Recruiting	Intravenous infusion	Patients with HCC; compares TACE with nivolumab alone or in combination with ipilimumab (26)
NCT03755791 (COSMIC-312)	Cabozantinib + Atezolizumab	TKI, PD-L1	Phase 3	Recruiting	Oral & IV infusion respectively	Advanced HCC, not received previous systemic therapy; compares efficacy versus sorafenib (370 combination treatment; 185 sorafenib; 185 cabozanitinib monotherapy)
NCT04246177(MK-7902-012/E7080- G000-318/LEAP-012)	Lenvatinib + Pembrolizumab	TKI, PD-1	Phase 3	Recruiting	Oral & IV infusion respectively	Incurable, non-metastatic HCC patients; compares efficacy of TACE with or without combination therapy (950)
NCT04777851 (RENOTACE)	Regorafenib + Nivolumab	TKI, PD-1	Phase 3	Not yet recruiting	Oral and IV, respectively	Intermediate stage HCC; compares TACE with combination therapy (496)
NCT03970616 (DEDUCTIVE)	Durvalumab + Tivozanib	PD-1, VEGF	Phase2/1	Recruiting	IV & oral respectively	Advance HCC patients (42)
NCT05312216	Lenvatinib + Durvalumab	TKI, PD-1	Phase 2	Not yet recruiting	Intravenous infusion	Unresectable HCC patients (25)
NCT04720716	Sintilimab + IBI310	PD-1, VEGF	Phase 3	Recruiting	Intravenous infusion	First line treatment for advanced HCC; compares efficacy versus sorafenib (490)
NCT03794440	Sintilimab + IBI305	PD-1, VEGF	Phase 2/3	Active, not recruiting	Intravenous infusion	First line treatment for advanced HCC; compares efficacy versus sorafenib (595)
NCT04465734	HLX10 + HLX04	PD-1, VEGF	Phase 3	Not yet recruiting	Intravenous infusion	First line treatment for locally advanced or metastatic HCC; compares efficacy versus sorafenib (477)
NCT04344158	AK105 + Anlotinib	PD-1, VEGF	Phase 3	Not yet recruiting	Intravenous infusion	Advanced HCC; compares efficacy versus sorafenib (648)
NCT04560894	SCI-I10A + SCT510	PD-1, VEGF	Phase 2/3	Recruiting	Intravenous infusion	Advanced HCC; compares efficacy versus sorafenib (621)
NCT03298451 (HIMALAYA)	Durvalumab + Tremelimumab	PD-1, CTLA-4	Phase 3	Recruiting	Intravenous infusion	Advance HCC patients; compares efficacy versus sorafenib or durvalumab monotherapy (1504)
NCT03778957 (EMERALD-1)	Durvalumab+ bevacizumab	PD-1, VEGF	Phase 3	Active, not recruiting	Intravenous infusion	Locoregional HCC not amenable to curative therapy; compares efficacy of TACE with durvalumab monotherapy or combination therapy (724)
NCT03847428 (EMERALD-2)	Durvalumab + bevacizumab	PD-1, VEGF	Phase 3	Recruiting	Intravenous infusion	High-risk of recurrence HCC after curative resection or ablation; compares efficacy with durvalumab monotherapy (888)
NCT02519348	Durvalumab + tremelimumab/durvalumab + bevacizumab	PD-1, CTLA-4, VEGF	Phase 2	Active, not recruiting	Intravenous infusion	Advanced HCC patients; compares efficacy with durvalumab or tremelimumab monotherapy (433)
Study identifier	Intervention	Target	Phase	Study status	Entry routes	Patients enrolled (enrolment target)
NCT04682210 (DaDaLi)	Sintilimab + apatinib	PD1, TKI	Phase 3	Not yet recruiting	Intravenous	HCC patients with high risk of recurrence after resection (246)
NCT03764293	SHR-1210 + apatinib	PD1, TKI	Phase 3	Active, not recruiting	Intravenous & oral, respectively	Locally advanced or metastatic and unresectable HCC patients; compares efficacy of combination versus sorafenib (543)
NCT04194775	CS1003 + lenvatinib	PD1, TKI	Phase 3	Recruiting	Intravenous	Advanced HCC patients not eligible for locoregional therapy; compares efficacy of combination versus lenvatinib (525)
NCT03605706	SHR-1210 + FOLFOX4	PD1, chemotherapy	Phase 3	Recruiting	Intravenous	Advanced HCC patients; compares efficacy of combination versus FOLFOX4 (396)
NCT04639180	Camrelizumab + apatinib	PD1, TKI,	Phase 3	Recruiting	Intravenous & oral, respectively	HCC patients with high risk of recurrence after resection or ablation (674)

**Table 5:** Data from https://www.clinicaltrials.gov/ accessed on 10 May 2022 showing clinical trials that use chimeric antigen T cell (CAR)-T cell therapy for the treatment of advanced hepatocellular carcinoma.

Study Identifier	Intervention	Phase	Study Status	Entry Routes	Patients Enrolled (Number of Patients)
NCT04121273	GPC3-CAR-T	Phase 1	Recruiting	Intravenous (IV) Infusion	GPC3 positive advanced HCC patients (20)
NCT03980288	GPC3-CAR-T	Phase 1	Completed	IV infusion	HCC patients (6)
NCT03146234	GPC3-CAR-T	Phase 1	Completed	IV infusion	Patients with relapse or refractory HCC (7)
NCT05003895	GPC3-CAR-T	Phase 1	Recruiting	IV infusion	Advance GPC3 expressing HCC patients (38)
NCT05155189	GPC3-CAR-T	Phase 1	Recruiting	IV infusion	Advance HCC patient (20)
NCT02395250	GPC3-CAR-T	Phase 1	Completed	IV infusion	Advance HCC patient (13)
NCT03884751	GPC3-CAR-T	Phase 1	Completed	IV infusion	Advanced HCC patients (9)
NCT05070156	GPC3-CAR-T	Phase 1	Recruiting	IV infusion	GPC3 positive advanced HCC patients (3)
NCT02905188	GPC3-CAR-T	Phase 1	Active, not recruiting	IV infusion	HCC patient (9)
NCT03198546	GPC3-CAR-T	Phase 1	Recruiting	IV infusion	HCC with GPC3 expression (30)
NCT05103631	IL-15+GPC3-CAR-T	Phase 1	Recruiting	IV infusion	GPC3-positive solid HCC tumor (27)
NCT03993743	CD147-CAR-T	Phase 1	Recruiting	Hepatic artery infusion	Very advanced HCC (34)
NCT03349255	Anti-HLA-A02/AFP-CAR-T	Phase 1	Terminated	IV infusion	AFP expressing HCC patients (3)
NCT03013712	EPCAM-CAR-T	Phase 1/2	Unknown	IV infusion	Advanced HCC (60)
NCT02729493	EPCAM-CAR-T	Phase 2	Unknown	IV infusion	Advanced HCC (25)
NCT05028933	EPCAM-CAR-T	Phase 1	Recruiting	IV infusion	Advanced HCC (48)
NCT02587689	Anti-MUC1 CAR-T	Phase 1/2	Unknown	IV infusion	Patients with MUC1 + advanced refractory solid tumor (20)
NCT05323201	B7H3 CAR-T	Phase 1	Recruiting	Transhepatic arterial infusion	Advanced B7H3-positive HCC (15)
NCT05131763	NKG2D-CAR-T	Phase 1	Recruiting	Hepatic portal artery injection	Patients with NKG2DL + solid tumor (3)
NCT04550663	NKG2D-CAR-T	Phase 1	Not yet recruiting	IV infusion	Relapsed or refractory NKG2DL + tumor (10)
